# Telocytes express PDGFRα in the human gastrointestinal tract

**DOI:** 10.1111/jcmm.12134

**Published:** 2013-09-24

**Authors:** Maria-Giuliana Vannucchi, Chiara Traini, Mirko Manetti, Lidia Ibba-Manneschi, Maria-Simonetta Faussone-Pellegrini

**Affiliations:** Department of Experimental and Clinical Medicine Section of Anatomy and Histology, University of FlorenceFlorence, Italy

**Keywords:** telocytes, human gastrointestinal tract, PDGFRα, CD34, c-kit, immunohistochemistry

## Abstract

Telocytes (TC), a cell population located in the connective tissue of many organs of humans and laboratory mammals, are characterized by a small cell body and extremely long and thin processes. Different TC subpopulations share unique ultrastructural features, but express different markers. In the gastrointestinal (GI) tract, cells with features of TC were seen to be CD34-positive/c-kit-negative and several roles have been proposed for them. Other interstitial cell types with regulatory roles described in the gut are the c-kit-positive/CD34-negative/platelet-derived growth factor receptor α (PDGFRα)-negative interstitial cells of Cajal (ICC) and the PDGFRα-positive/c-kit-negative fibroblast-like cells (FLC). As TC display the same features and locations of the PDGFRα-positive cells, we investigated whether TC and PDGFRα-positive cells could be the same cell type. PDGFRα/CD34, PDGFRα/c-kit and CD34/c-kit double immunolabelling was performed in full-thickness specimens from human oesophagus, stomach and small and large intestines. All TC in the mucosa, submucosa and muscle coat were PDGFRα/CD34-positive. TC formed a three-dimensional network in the submucosa and in the interstitium between muscle layers, and an almost continuous layer at the submucosal borders of muscularis mucosae and circular muscle layer. Moreover, TC encircled muscle bundles, nerve structures, blood vessels, funds of gastric glands and intestinal crypts. Some TC were located within the muscle bundles, displaying the same location of ICC and running intermingled with them. ICC were c-kit-positive and CD34/PDGFRα-negative. In conclusion, in the human GI tract the TC are PDGFRα-positive and, therefore, might correspond to the FLC. We also hypothesize that in human gut, there are different TC subpopulations probably playing region-specific roles.

## Introduction

Telocytes (TC), formerly called interstitial Cajal-like cells (ICLC), are a type of interstitial cells recently identified in the connective tissue of many organs of humans and laboratory mammals [1–9]. These cells are characterized by a small nucleated body and 2–3 extremely long, thin and varicose processes [3, 6]. To date, an unequivocal identification of TC can be made only under transmission electron microscope (TEM), as by immunohistochemistry these cells do not express specific markers and, according to the organ and/or the animal species examined, some of them were found to be c-kit-positive, some CD34-positive, some others were reported to express vimentin, caveolin-1, iNOS and VEGF [see Ref. [6] for review]. Thus, it has been proposed that several TC subpopulations sharing the same ultrastructural features but displaying different immunophenotypes may exist, and it has been suggested that such differences might be at the basis of region-specific TC roles [4, 6].

To date, three different interstitial cell types have been identified in the gastrointestinal (GI) tract, each forming networks within the muscle coat:

- The interstitial cells of Cajal (ICC) that are positive for c-kit and negative for CD34 and platelet-derived growth factor receptor α (PDGFRα) [2, 10–17]. These cells are considered the pacemaker cells and the mediators of neurotransmission in the GI tract [10, 12, 17, 18].
- The TC that are CD34-positive and c-kit-negative [2]. Several roles have been proposed for these cells. In particular, in the gut, their three-dimensional (3-D) networks might play a mechanical and supporting role being resistant to and deformable following intestine movements [2]. Moreover, as some of the intramuscular TC and ICC seem to be part of a unique network, in which only/preferentially ICC are in close contact with nerve endings, these TC might play a role in neurotransmission, possibly contributing to spread the slow waves generated by the ICC [2].
- The PDGFRα-positive and c-kit-negative cells, previously called ‘fibroblast-like’ cells (FLC) [19–25]. The PDGFRα-positive cells have been suggested to play an important role in enteric neurotransmission, in particular in the purinergic and the inhibitory ones [21–23].

Furthermore, it has been reported that in the murine and human GI muscle coat, PDGFRα-positive cells express the small-conductance calcium-activated potassium channel type 3 (SK3) [19, 23, 24]. In humans, guinea pig and mouse, SK3-positive cells express CD34 but not c-kit, and therefore these cells are not ICC [24, 26–30]. These data are of interest as SK3 is involved in the control of neuronal excitability and has potential functional significance in the GI muscle coat [24].

Briefly, coordinated GI motor activity requires a complex interplay among various cell types including inhibitory and excitatory neurons, smooth muscle cells, ICC and other interstitial cell types. From all the above mentioned data, it can be concluded that in the GI tract there are CD34-, SK3- and PDGFRα-positive interstitial cells organized in networks similar to those formed by the ICC, from which, however, they are definitely distinct. However, it has to be demonstrated whether the CD34-, SK3- and PDGFRα-positive interstitial cells are a unique cell type probably corresponding to the TC, which have been identified as CD34-positive interstitial cells [2].

On this basis, the present study was designed to investigate whether the TC and the PDGFRα-positive cells could be the same interstitial cell type throughout different segments of the human gut, including the oesophagus, gastric fundus, corpus and antrum, small and large intestine. The findings obtained will allow establishing how many types of interstitial cells are present in the human gut. Moreover, the identification of a functional receptor, such as the PDGFRα, on the TC might help to better understand the role(s) played by these cells in the human GI tract.

## Materials and methods

Full-thickness samples from distal oesophagus (patients *n* = 4, aged between 39 and 66 years), stomach (fundus, corpus and antrum, *n* = 16, aged between 35 and 71 years), small intestine (patients *n* = 7, aged between 41 and 72 years) and large intestine (ascending, right and left transverse and descending colon, patients *n* = 16, aged between 48 and 75 years) were obtained from patients undergoing surgery for cancer. Care was taken in selecting specimens far from the tumour, 5 cm or more away from the site of cancer, and in choosing the areas devoid of inflammation. The patients had not taken any drugs affecting gut motility. All the patients signed a written informed consent form and the local ethics committee approved the study. Specimens were processed for immunofluorescence and immunohistochemistry. Immediately after surgery, all the specimens were fixed in 10% buffered formalin, dehydrated in a graded ethanol series, cleared in xylene and embedded in paraffin. The sections were cut (5 μm thick) by using a rotary microtome (MR2, Boeckeler Instruments Inc., Tucson, AZ, USA; or Leica RM2255, Leica Microsystems, Mannheim, Germany), deparaffinized in xylene followed by graded ethanol series and stained with haematoxylin/eosin for routine histology.

### Immunofluorescence

For antigen retrieval, the sections were deparaffinized and boiled for 10 min. in sodium citrate buffer (10 mM, pH 6.0, Bio-Optica, Milan, Italy) or treated for 20 min. at 90–92°C with Tris (10 mM/l) and EDTA (1 mM/l, pH 9.0) buffer, as appropriate. The sections were washed in PBS (0.1 M), incubated in 2 mg/ml glycine (AppliChem, Darmstadt, Germany) for 10 min. to quench autofluorescence caused by free aldehydes, and then blocked for 20–45 min. at room temperature (RT) with 1% bovine serum albumin (BSA, Sigma-Aldrich, St. Louis, MO, USA) in PBS. For the double immunofluorescent staining, the sections were incubated overnight (O/N) at 4°C with a mixture of primary antibodies diluted in PBS with 1% BSA. Information on primary antibody sources and concentrations is shown in Table 1. The day after, the slides were washed three times in PBS and incubated for 45 min./2 hrs at RT in the dark with a mixture of appropriate fluorochrome-conjugated secondary antibodies (Alexa Fluor 488-conjugated, Alexa Fluor 568-conjugated, or Rhodamine Red-X-conjugated IgG; Invitrogen, San Diego, CA, USA) diluted 1:200 in PBS with 1% BSA. Irrelevant isotype- and concentration-matched IgG (Sigma-Aldrich) were used as negative controls. Cross-reactivity of secondary antibodies was tested in control experiments in which primary antibodies were omitted. Some slides were counterstained with 4′,6-diamidino-2-phenylindole (DAPI; Chemicon International, Temecula, CA, USA). Tissue sections were then thoroughly washed in PBS, mounted in an aqueous medium (Fluoremount, Sigma-Aldrich) and observed under an epifluorescence Zeiss Axioskop microscope (Zeiss, Oberkochen, Germany) or a Leica DM4000 B microscope equipped with fully automated transmitted light and fluorescence axes (Leica Microsystems). Fluorescence images were captured using a Leica DFC310 FX 1.4-megapixel digital colour camera equipped with the Leica software application suite LAS V3.8 (Leica Microsystems).

**Table 1:** Details of primary antibodies

Primary antibody	Host	Antigen retrieval	Working dilution	Source
PDGFRα	Goat	Sodium citrate buffer, pH 6.0 Tris-EDTA buffer, pH 9.0	1:100	R&D Systems, Minneapolis, MN, USA; Catalogue no. AF-307-NA
CD34	Mouse	Sodium citrate buffer, pH 6.0 Tris-EDTA buffer, pH 9.0	1:50	Dako, Glostrup, Denmark; Catalogue no. M7165
c-Kit	Rabbit	Tris-EDTA buffer, pH 9.0	1:300	Dako, Glostrup, Denmark; Catalogue no. A4502
CD31/PECAM-1	Rabbit	Sodium citrate buffer, pH 6.0	1:50	Abcam, Cambridge, UK; Catalogue no. ab28364
PGP9.5	Rabbit	Sodium citrate buffer, pH 6.0	1:200	Chemicon-Millipore, Temecula, CA; Catalogue no. AB1761

### Immunohistochemistry

After deparaffinization and antigen retrieval phase in sodium citrate buffer (10 mM, pH 6.0, Bio-Optica), the sections were treated with 3% H_2_O_2_ in PBS for 5 min. to block endogenous peroxidase activity. After washing, the sections were incubated in 2 mg/ml glycine for 10 min. and then with 1% BSA in PBS for 20 min. at RT. The primary antibodies, anti-PDGFRα or anti-CD34, diluted in PBS were applied O/N at 4°C. The day after, the sections were incubated with a polyclonal biotinylated secondary antibody diluted in PBS for 2 hrs at RT. Subsequently, the sections were washed, treated with ABC Reagent (Vectastain ABC/Elite Kit, Vector Laboratories, Burlingame, CA, USA) for 20 min. and developed with 3,3′-diaminobenzidine (DAB, Sigma-Aldrich) or 3-amino-9-ethylcarbazole (AEC kit, LabVision, Fremont, CA, USA) as chromogen. Some sections were counterstained with haematoxylin. After washing in PBS, the sections were mounted in an aqueous medium (Sigma-Aldrich) and observed under the Leitz Axioscop or the Leica DM4000 B microscope.

Sections not exposed to the primary antibodies were included as negative controls for antibody specificity. Cross-reactivity of secondary antibodies was tested in negative controls performed omitting the primary antibodies.

## Results

### PDGFRα immunolabelling

**Fig. 1:**
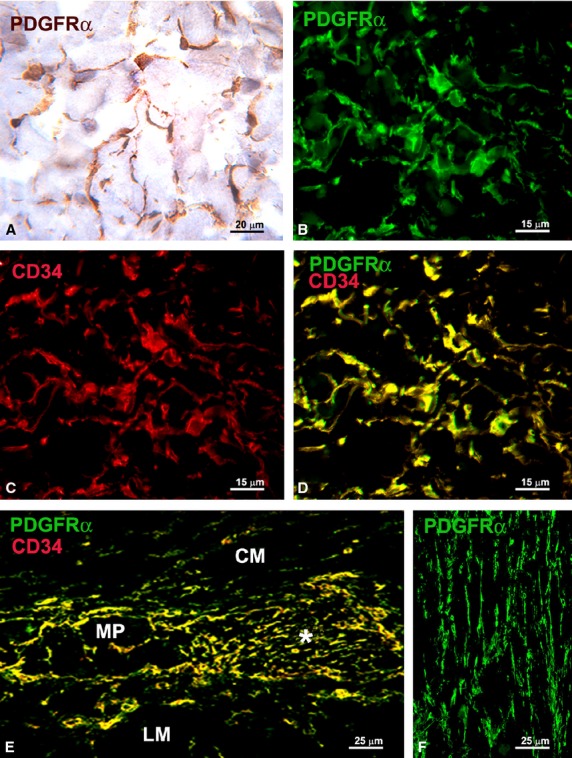
(**A, B** and **F**) PDGFRα-immunoreactivity; (**C**) CD34-immunoreactivity; (**D** and **E**) PDGFRα/CD34 double labelling. (**A**) Immunohistochemistry, haematoxylin counterstain; (**B**–**F**) Immunofluorescence. (**A**–**D**) Submucosa (stomach). PDGFRα-positive cells (**A** and **B**) and CD34-positive cells (**C**) form a 3-D network. All the PDGFRα-positive cells are also CD34-positive (**D**). (**E**) Myenteric plexus region (large intestine). PDGFRα/CD34-positive cells surround a ganglion (left side, MP) and form networks in the intergangliar region (right side, asterisk). (**F**) Circular muscle layer (small intestine). PDGFRα-positive cells form networks among the smooth muscle cells. CM: circular muscle layer; LM: longitudinal muscle layer. Scale bars are indicated in each panel.

**Fig. 2:**
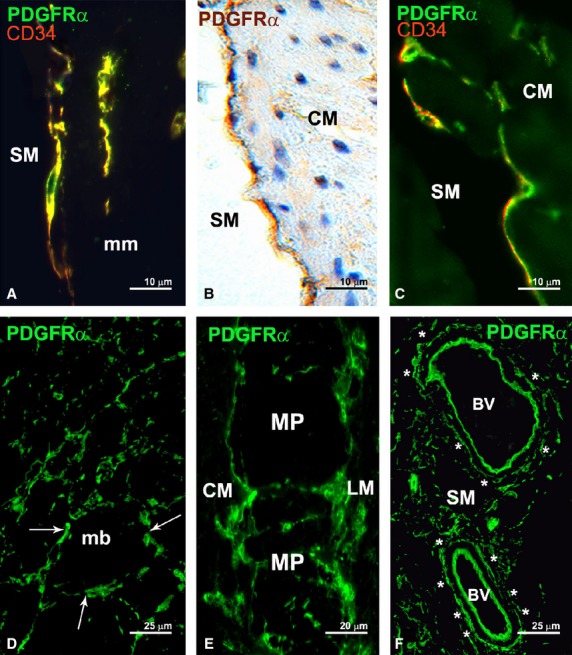
(**A** and **C**) PDGFRα/CD34 double labelling; (**B** and **D**–**F**) PDGFRα-immunoreactivity. (**A** and **C**–**F**) Immunofluorescence; (**B**) Immunohistochemistry, haematoxylin counterstain. PDGFRα-positive cells at the submucosal borders of the muscularis mucosae (**A**, stomach) and circular muscle layer (**B** and **C**, stomach). PDGFRα-positive cells also show CD34-immunoreactivity (**A** and **C**). (**D**) Circular muscle layer (oesophagus). PDGFRα-positive cells border the smooth muscle bundles (arrows). (**E**) Myenteric plexus region (small intestine). PDGFRα-positive cells surround the ganglia. (**F**) Submucosa (small intestine). PDGFRα-positive cells encircle large blood vessels (asterisks). BV: blood vessel; CM: circular muscle layer; LM: longitudinal muscle layer; mb: muscle bundles; mm: muscularis mucosae; MP: ganglia at the myenteric plexus; SM: submucosa. Scale bars are indicated in each panel.

**Fig. 3:**
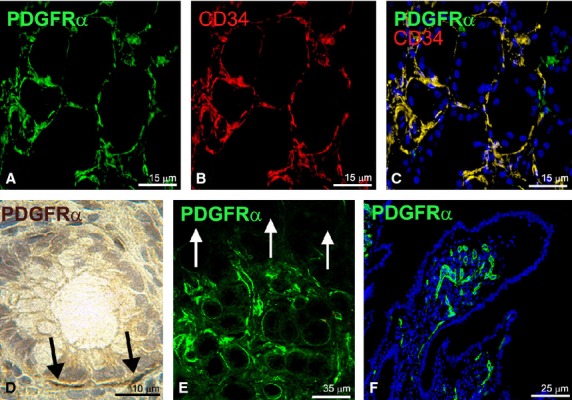
(**A** and **D**–**F**) PDGFRα-immunoreactivity; (**B**) CD34-immunoreactivity; (**C**) PDGFRα/CD34 double labelling. (**A**–**C, E** and **F**) Immunofluorescence (nuclei are blue stained with DAPI in (**C** and **F**); (**D**) Immunohistochemistry, haematoxylin counterstain. (**A**–**C**) Mucosa (stomach). Numerous PDGFRα/CD34-positive cells surround the funds of the glands. (**D**) Mucosa (large intestine). Few and scattered PDGFRα-positive cells surround glandular crypts (black arrows). (**E**) Mucosa (stomach). PDGFRα-positive cells are numerous around funds of the glands and absent from the superficial mucosa (indicated by white arrows). (**F**) Mucosa (small intestine). Only endothelial cells of capillary vessels display PDGFRα-immunoreactivity. Scale bars are indicated in each panel.

**Fig. 4:**
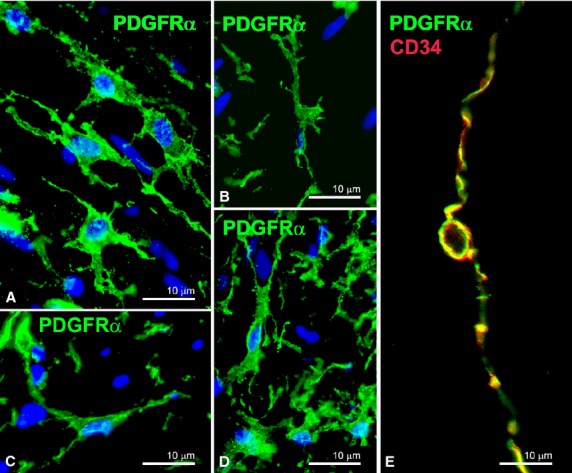
(**A**–**D**) PDGFRα-immunoreactivity (nuclei are blue stained with DAPI); (**E**) PDGFRα/CD34 double labelling. (**A** and **B**) Muscle layers (small intestine). Intramuscular PDGFRα-positive cells display two long telopodes and several short processes starting from the nucleated portion. (**C**) Submucosa (small intestine). PDGFRα-positive cells show a triangular body and three long and varicose telopodes. (**D**) Myenteric plexus region (small intestine). PDGFRα-positive cells display an oval body and several telopodes running in every direction. (**E**) A PDGFRα/CD34-positive cell at the border of a circular muscle bundle (small intestine) shows a small nucleated body and two long and thin telopodes starting from the opposite poles of the cell and with podomers and podoms clearly identifiable. Scale bars are indicated in each panel.

Platelet-derived growth factor receptor α -positive cells were seen, both under light and fluorescence microscopes, to form 3-D networks in the submucosa and between the two muscle layers throughout the entire GI tract (Fig. 1A, B, D and E). Several PDGFRα-positive cells, apparently forming networks, were also located among the smooth muscle cells (Fig. 1F). An almost continuous monolayer of PDGFRα-positive cells was present at the submucosal borders of the muscularis mucosae and of the circular muscle layer (Fig. 2A–C). Moreover, PDGFRα-positive cells bordered the smooth muscle bundles of both muscle layers (Fig. 2D) and surrounded the enteric nerve strands and ganglia (Figs 1E and 2E), as well as large blood vessels (Fig. 2F). Some PDGFRα-positive cells were seen in the mucosa surrounding the funds of the gastric glands and the intestinal crypts (Fig. 3A, C–E). In particular, those in the gastric mucosa were numerous, while those in the mucosa of small and large intestine were few and scattered. No PDGFRα-positive interstitial cell was seen within the superficial mucosa (Fig. 3F; see also Fig. 7E). All these PDGFRα-positive cells displayed the characteristic features of the TC: a slender nucleated body and few long, thin and varicose processes (telopodes with alternating podomers and podoms) [3, 6] (Fig. 4). Some of the features of the PDGFRα-positive cells, however, varied according to their location. Most of them, in particular those surrounding the funds of the gastric glands and the intestinal crypts, muscularis mucosae, muscle layers, ganglia and blood vessels, had a small oval body and two telopodes starting from the opposite cell poles with alternating podomers and podoms clearly identifiable (Figs 2A–F, 3A, C–E and 4E). The intramuscular ones, although frequently displaying two long telopodes running parallel to the major axis of the smooth muscle cells, had also several short processes starting from the nucleated portion and running transversely around the smooth muscle cells (Figs 1F and 4A, B). Moreover, most of the submucosal PDGFRα-positive cells had a triangular or polygonal body and at least three very long and varicose processes (Figs 1A, B, D and 4C). Finally, those at the myenteric plexus region displayed a large oval or roundish body and 3–4 processes running in every direction and contacting each other (Figs 1E and 4D).

### CD34 immunolabelling

**Fig. 5:**
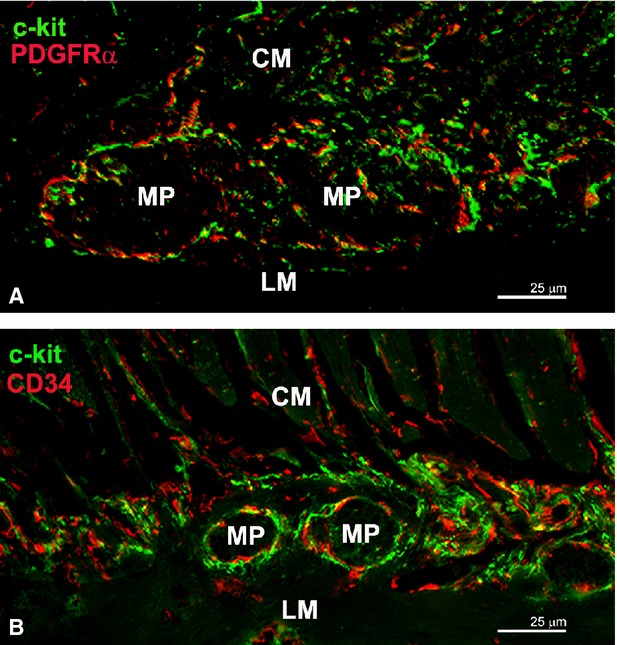
(**A**) PDGFRα/c-kit double labelling; (**B**) CD34/c-kit double labelling. Myenteric plexus region (small intestine). All the PDGFRα- and CD34-positive cells (telocytes) are c-kit-negative. Interstitial cells of Cajal (ICC) are c-kit-positive. Some telocytes located within the muscle bundles and at the myenteric plexus region show the same location of ICC and are intermingled with them. Their close spatial relationships raise the presence of yellow spots. CM: circular muscle layer; LM: longitudinal muscle layer; MP: ganglia at the myenteric plexus. Scale bars are indicated in each panel.

**Fig. 6:**
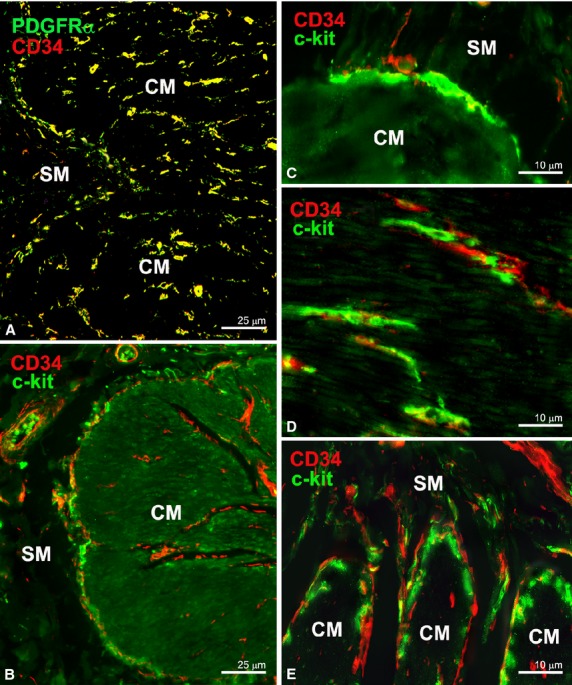
(**A**) PDGFRα/CD34 double labelling; (**B**–**E**) CD34/c-kit double labelling. All the PDGFRα-positive cells (**A**, large intestine) in the submucosa, at the submucosal border of the circular muscle layer and in the muscle wall are also CD34-positive. (**B**; large intestine and **C**; stomach): Interstitial cells of Cajal (ICC) at the submucosal border of the circular muscle layer (ICC-SM). (**D**; large intestine): Intramuscular ICC (ICC-IM). (**E**; small intestine): ICC at the deep muscular plexus (ICC-DMP). All the ICC are c-kit-positive and CD34-negative. At the submucosal border of the circular muscle layer, the CD34-positive cells (telocytes) are located close to the submucosa while the ICC-SM are closer to the circular smooth muscle cells (**B** and **C**). Several ICC-IM are intermingled with telocytes (**D**). The ICC-DMP appear to be not accompanied by telocytes (**E**). CM: circular muscle layer; SM: submucosa. Scale bars are indicated in each panel.

CD34-positive cells, similar to the PDGFRα-positive cells, were distributed around and within the circular and longitudinal muscle layers, in the myenteric plexus region, in the mucosa, submucosa and muscularis mucosae (Figs 1 C–E, 2A, C, 3B, C, 4E, 5B and 6A). These CD34-positive cells showed a slender cell body with long and thin processes, and, as previously observed for the PDGFRα-positive cells, their shape varied according to the cell location.

### c-kit immunolabelling

All the cells identifiable as ICC for their shape and region-specific location [10–17], *i.e*. the ICC located in the region around myenteric ganglia and between the two muscle layers (ICC-MP; Fig. 5A and B), the ICC at the submucosal border of the gastric and colonic circular muscle layer (ICC-SM; Fig. 6B and C), the intramuscular ICC (ICC-IM; Fig. 6D), and the ICC at the deep muscular plexus (ICC-DMP; Fig. 6E), were c-kit-positive.

### Double immunolabelling

**Fig. 7:**
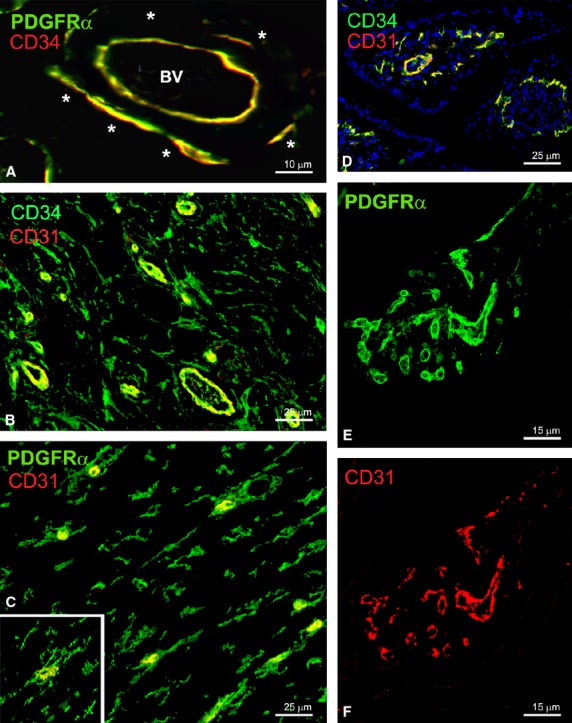
(**A**; submucosa, large intestine): Endothelial cells of blood vessels and perivascular telocytes (asterisks) are PDGFRα/CD34-positive. (**B**; submucosa, large intestine): CD34/CD31 double labelling. (**C**; circular muscle layer, large intestine): PDGFRα/CD31 double labelling. Endothelial cells are CD31/CD34/PDGFRα-positive while telocytes are CD31-negative. To note that telocytes are close to blood capillaries (**C** and inset). (**D**–**F**) Villous stroma (small intestine). Endothelial cells are positive for CD31 (**D** and **F**), CD34 (**D**) and PDGFRα (**E**). Nuclei are blue stained with DAPI in **D**. BV: blood vessel. Scale bars are indicated in each panel.

All the PDGFRα-positive cells in the muscle wall, in the submucosa and mucosa were also CD34-positive and *vice versa* (Figs 1D, E, 2A, C, 3C, 4E, 6A and 7A); none of them was c-kit-positive (Fig. 5A). These CD34/PDGFRα-positive cells had the typical features and locations of TC, allowing us to conclude that all of them are TC. Therefore, hereafter these cells will be referred to as TC. Conversely, all the ICC were CD34/PDGFRα-negative (Figs 5A, B and 6B–E). Of note, TC and ICC were often in close proximity to each other. However, while some of the TC located within the muscle bundles and at the myenteric plexus region had the same location of the ICC-IM and the ICC-MP, respectively, and were frequently intermingled with them (Figs 5A, B and 6D), no TC was observed to intermingle with the ICC-DMP (Fig. 6E). At the submucosal border of both gastric and colonic circular muscle layer, the TC were located close to the submucosa while the ICC-SM were closer to the circular smooth muscle cells (Fig. 6B and C).

The endothelial cells of the blood vessels, similar to the TC, were PDGFRα- and CD34-positive throughout the entire GI tract (Fig. 7A and E). However, endothelial cells could be clearly distinguished from the TC because only endothelial cells expressed the pan-endothelial cell marker CD31/PECAM-1 (Fig. 7B–F). To note, the TC, especially those within the muscle layers, were frequently close to the blood capillaries (Fig. 7C and inset).

**Fig. 8:**
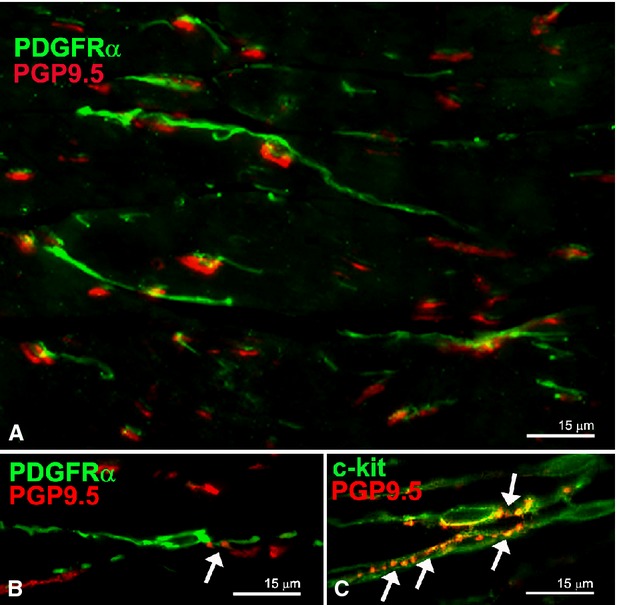
(**A** and **B**) PDGFRα/PGP9.5 double labelling; **C**: c-kit/PGP9.5 double labelling. (**A**–**C**) Circular muscle layer (stomach). Close, although occasional, relationships between nerve fibres and intramuscular telocytes (arrow in **B**). Numerous close contacts between nerve endings and ICC-IM (arrows in **C**). Scale bars are indicated in each panel.

Finally, PDGFRα/PGP9.5 double labelling revealed that nerve fibres and intramuscular TC were occasionally close to each other (Fig. 8A and B). Conversely, c-kit/PGP9.5 double labelling showed numerous close contacts between nerve endings and ICC (Fig. 8C).

## Discussion

In the entire human GI tract, PDGFRα-positive cells were organized to form 3-D networks in the submucosa and in the interstitium between the longitudinal and circular muscle layers. Moreover, these cells formed an almost continuous monolayer at the submucosal borders of the muscularis mucosae and circular muscle layer, encircled the muscle bundles and surrounded ganglia, nerve strands, blood vessels, funds of gastric glands and intestinal crypts. These locations are identical to those we have previously described for the CD34-positive cells in the human stomach, ileum and colon [2]. Double labelling clearly demonstrated a co-localization of PDGFRα- and CD34-immunoreactivity. Therefore, we can conclude that in the human GI tract, PDGFRα-positive and CD34-positive cells are the same cell type. As by immuno-light, -fluorescence and -electron microscopy we previously showed that in the human GI tract the CD34-positive cells are TC [2], we can presently affirm that TC express PDGFRα. In support, the shape of the TC, as previously reported [3, 6], is identical to that we have seen for the PDGFRα/CD34-positive cells. Moreover, according to literature, in the human gut both the CD34-positive [26–28] and the PDGFRα-positive cells express SK3 [23, 24]. Therefore, we can reasonably conclude that TC might be also SK3-positive.

Differently from the study of Grover *et al*. [24], who reported a greater PDGFRα-positive cell density in the longitudinal muscle layer of human gastric corpus when compared with the circular one, we could not appreciate differences in cell density between the two muscle layers in any gut region. However, we should consider that some precautions are necessary when identifying TC by immunolabelling. Indeed, endothelial cells also express both CD34 and PDGFRα and blood capillary profiles could sometimes be mistaken with TC. As we presently reported, the combination of CD34 or PDGFRα immunolabelling with an endothelial cell-specific marker (*i.e*., CD31/PECAM-1) may be helpful to unequivocally identify TC. Additionally, autofluorescent elastic fibres, that are particularly abundant in the different layers of the human GI wall, could be mistaken with TC. DAPI counterstaining for nuclei allowed us to distinguish between TC and elastic fibres.

Consistent with previous studies [2, 19, 20, 22–27], we found that all the ICC populations were c-kit-positive and PDGFRα/CD34-negative. Therefore, we can confirm that ICC represent a specific interstitial cell type unequivocally distinct from TC. As already reported [23, 24], here we show that the TC located within the muscle bundles and at the myenteric plexus region share the same location of the ICC. Besides, TC processes appeared to form networks intermingling with those of ICC, suggesting that these two cell types might establish cell-to-cell contacts. We also noted that some TC had a distribution distinct from that of ICC. In fact, no TC appeared intermingled with the ICC-DMP and, at the submucosal border of both gastric and colonic circular muscle layer, TC were located close to the submucosa, while the ICC-SM were close to the circular smooth muscle cells.

Recent studies on human and murine GI tract [19, 25] reported PDGFRα-positive cells in the mucosa. Kurahashi *et al*. [25] observed that these cells have a different labelling intensity according to their specific mucosal location and demonstrated that none of them was either myofibroblasts, or fibroblasts or smooth muscle cells. In agreement with these studies, we found PDGFRα-positive cells in the mucosa and, therefore, we can identify these cells as TC as they were also CD34-positive. In support to this conclusion, the presence of TC throughout the entire GI mucosa has previously been demonstrated by electron microscopy [9, 30–32]. Of note, differently from these findings [9, 19, 25, 30–32], in our specimens only the TC located around the basal portion of the glands were PDGFRα/CD34-positive. This discrepancy might be as a result of the different tissue fixation and embedding methodologies employed. However, there is also the possibility that the TC have different PDGFRα and CD34 staining affinities according to their location in the entire wall of the GI tract.

The possible relationship between the PDGFRα-positive cells and nerve fibres in the gut muscle coat has been the subject of great attention. It has been reported that the PDGFRα-positive cells (formerly called FLC) express nitric oxide-sensitive soluble guanylate cyclase in the guinea pig GI tract, SK3 and purinergic receptors in the murine intestine and in the muscle coat of human stomach and colon, and that they are closely related to ICC, nNOS- and SP-positive nerve fibres [21–24, 33, 34]. On this basis, it has been suggested that the PDGFRα-positive cells might have a potential role in neurotransmission, especially in the inhibitory one [21, 23]. We presently observed that in the submucosa, the TC and the nerve fibres ran independently from each other. Within the muscle layers, TC and ICC were intermingled and formed a unique network running together with the nerve fibres. However, only some nerve endings were in proximity to TC, while numerous close contacts were present between ICC and nerve endings. These findings support our previous hypothesis [2] that the intramuscular TC might play a role in neurotransmission, possibly contributing to spread the slow waves generated by the ICC. Further functional evidence is required to fully ascertain whether the nerve fibres close to the intramuscular TC and ICC influence both of them or only/preferentially the ICC.

At present, in spite of the growing literature on the TC, it is still difficult to identify their role(s). Some of the roles previously suggested for the GI TC can be re-proposed [2]: (*i*) the TC 3-D network at the myenteric plexus region and in the submucosa might be resistant to and deformable following the intestinal movements to guarantee the integrity of the tissue during stretching; (*ii*) the TC at the submucosal border of the circular muscle layer might play a mechanical, supporting role and/or, as in the myocardium, create microenvironments within the tissue [2, 35, 36]. In this context, the present finding demonstrating that TC express the functional receptor PDGFRα might help in identifying further role(s). Indeed, it has been reported that PDGF/PDGFR signalling plays critical roles in mammalian organogenesis and murine GI villous morphogenesis, and it has been demonstrated that selective inhibition of PDGFR suppresses longitudinal smooth muscle differentiation [37–40]. Therefore, the expression of PDGFRα might be implicated in the function proposed for TC as nurse cells for stem cell niches [41–44]. In this regard, the PDGFRα-positive TC observed around the basal portion of the glands, where epithelial stem cells are located [45], is in agreement with this potential role. In addition, the TC, occupying an important niche in the lamina propria, might function in the transduction of sensory and immune signals and in the maintenance of mucosal homoeostasis [45]. According to our previous hypothesis [2], TC might also represent the adult stromal mesenchymal cells. The expression of PDGFRα by TC reinforces such hypothesis as it is known that PDGFRα is expressed in mesenchymal stem cells [46]. Therefore, the maintenance of PDGFRα in adulthood is consistent with the possible TC capability to differentiate in different cell lines, when necessary.

To date, the majority of studies have investigated TC in physiological conditions. Interestingly, it has been reported that SK-3 channels were present in the myometrial TC [47] and in skeletal muscle interstitium, TC appeared to be PDGFRβ-positive [48]. In our opinion, future studies on different pathologies will help shed light on the possible TC functions. Recently, TC have been reported to be directly involved in skin alterations of patients affected by scleroderma [49], and the presence of PDGFRα on TC may have an important role in the pathophysiology of several organs [50–52]. In the context of GI tract pathophysiology, the possible involvement of TC has been investigated in human gastroparesis, but no changes in shape, location and number of PDGFRα-positive cells were reported [24].

In conclusion, our data indicate that in human GI tract TC express PDGFRα. Therefore, these TC and the PDGFRα-positive cells (also called FLC by several authors) are the same cell type. Moreover, on the basis of the TC location and different PDGFRα staining affinities within the GI wall, we hypothesize that in human, gut there are several TC subpopulations probably playing region-specific roles.
